# Localized Connectivity in Obsessive-Compulsive Disorder: An Investigation Combining Univariate and Multivariate Pattern Analyses

**DOI:** 10.3389/fnbeh.2019.00122

**Published:** 2019-06-13

**Authors:** Xinyu Hu, Lianqing Zhang, Xuan Bu, Hailong Li, Bin Li, Wanjie Tang, Lu Lu, Xiaoxiao Hu, Shi Tang, Yingxue Gao, Yanchun Yang, Neil Roberts, Qiyong Gong, Xiaoqi Huang

**Affiliations:** ^1^Huaxi MR Research Center (HMRRC), West China Hospital of Sichuan University, Chengdu, China; ^2^Mental Health Center, West China Hospital of Sichuan University, Chengdu, China; ^3^Clinical Research Imaging Centre (CRIC), The Queen’s Medical Research Institute (QMRI), University of Edinburgh, Edinburgh, United Kingdom

**Keywords:** obsessive-compulsive disorder, resting-state functional magnetic resonance imaging, psychoradiology, localized connectivity, regional homogeneity, univariate analysis, multivariate pattern analysis, support vector machine

## Abstract

Recent developments in psychoradiological researches have highlighted the disrupted organization of large-scale functional brain networks in obsessive-compulsive disorder (OCD). However, whether abnormal activation of localized brain areas would affect network dysfunction remains to be fully characterized. We applied both univariate analysis and multivariate pattern analysis (MVPA) approaches to investigate the abnormalities of regional homogeneity (ReHo), an index to measure the localized connectivity, in 88 medication-free patients with OCD and 88 healthy control subjects (HCS). Resting-state functional magnetic resonance imaging (RS-fMRI) data of all the participants were acquired in a 3.0-T scanner. First, we adopted a traditional univariate analysis to explore ReHo alterations between the patient group and the control group. Subsequently, we utilized a support vector machine (SVM) to examine whether ReHo could be further used to differentiate patients with OCD from HCS at the individual level. Relative to HCS, OCD patients showed lower ReHo in the bilateral cerebellum and higher ReHo in the bilateral superior frontal gyri (SFG), right inferior parietal gyrus (IPG), and precuneus [*P* < 0.05, family-wise error (FWE) correction]. ReHo value in the left SFG positively correlated with Yale–Brown Obsessive Compulsive Scale (Y-BOCS) total score (*r* = 0 0.241, *P* = 0.024) and obsessive subscale (*r* = 0.224, *P* = 0.036). The SVM classification regarding ReHo yielded an accuracy of 78.98% (sensitivity = 78.41%, specificity = 79.55%) with *P* < 0.001 after permutation testing. The most discriminative regions contributing to the SVM classification were mainly located in the frontal, temporal, and parietal regions as well as in the cerebellum while the right orbital frontal cortex was identified with the highest discriminative power. Our findings not only suggested that the localized activation disequilibrium between the prefrontal cortex (PFC) and the cerebellum appeared to be associated with the pathophysiology of OCD but also indicated the translational role of the localized connectivity as a potential discriminative pattern to detect OCD at the individual level.

## Introduction

Obsessive-compulsive disorder (OCD) is among the most debilitating mental illnesses that influence nearly 2.3% of the general population (Abramowitz et al., 2009). In spite of the high morbidity as well as high levels of social burden (Ruscio et al., 2010), the pathological mechanisms of OCD remain elusive. The investigation of the neurobiological substrates of OCD is a fundamental point to shed light upon the underlying mechanisms, which may be of great value in gaining insights into improving the specificity of diagnosis and efficacy of treatment for OCD.

Over the past decade, advanced magnetic resonance imaging (MRI) techniques have dramatically facilitated the comprehension of the neural correlates in OCD (Nakao et al., 2014). Previous review of voxel-based morphometry studies reported gray matter alterations of both “affective” and “executive” circuits in OCD patients (Piras et al., 2015). Meta-analysis of diffusion MRI literature consistently showed microstructural alterations of the fronto-basal pathways and intra-hemispheric bundles in OCD (Piras et al., 2013). Meanwhile, aberrant structural connectivity of left superior longitudinal fasciculus and the body of corpus callosum has been identified in patients with OCD, which was associated with executive control function (Spalletta et al., 2014). For functional MRI (fMRI) researches in OCD, patients show abnormal activation in several brain regions, which are essential for some domains of neuropsychological function such as decision making [ventromedial orbitofrontal cortex (OFC)] (Norman et al., 2018), error monitoring [amygdala, presupplementary motor area (preSMA), and subgenual anterior cingulate cortex] (Grützmann et al., 2016), response inhibition [inferior parietal gyrus (IPG), inferior frontal gyrus, and preSMA] (de Wit et al., 2012), reward-based learning (hippocampus, putamen, and amygdala; Marsh et al., 2015), fear conditioning (caudate and hippocampus) and extinction recall (cerebellum, posterior cingulate cortex, and putamen; Milad et al., 2013), cognitive flexibility [caudate and ventrolateral prefrontal cortex (PFC)], as well as goal-directed planning (putamen and dorsolateral PFC; Vaghi et al., 2017). The results obtained from these functional neuroimaging researches appear to be highly inconsistent, which might be attributed to clinical heterogeneity (e.g., symptom severity, onset age, illness duration, medication exposure, and comorbidity profiles) of OCD participants. More importantly, findings from task-based neuroimaging studies varied remarkably as different paradigms or analytic methods were adopted.

Resting-state fMRI (RS-fMRI) is a popular psychoradiological approach to assess the brain function at rest without performing a task (Lui et al., 2016). Two derived RS-fMRI parameters including the amplitude low-frequency flow-frequency (ALFF) and functional connectivity (FC) are widely adopted to explore the cerebral dysfunction in mental disorders. ALFF reflects the intensity of spontaneous neural activity while the FC reflects the level of integration of local activity across brain regions (Biswal et al., 1995; Gusnard and Raichle, 2001). Besides applying these two methods to explore the regional and network-level neural function of the brain, we can alternatively characterize the local synchronization of spontaneous blood oxygen level-dependent (BOLD) signal fluctuation among neighboring voxels within a given cluster, using an index named as regional homogeneity (ReHo; Zang et al., 2004). Given the hypothesis that similar temporal patterns are shared by spatially neighboring voxels, Kendall’s coefficient of concordance (KCC) between the time series of a single voxel and those of its neighbors was calculated in the ReHo analysis using a voxel-wise manner. Considering the computational basis of this parameter, ReHo could be best described as an index of “localized connectivity,” which gives the researchers a chance to discover the disruptions of localized activation in disease states without *a priori* constraints and makes it possible to investigate the previously unconsidered regional alterations (Iwabuchi et al., 2015).

Previous RS-fMRI studies using conventional mass-univariate analytical techniques for investigating the alterations of ReHo in OCD have suggested that there may be anomalies of localized connectivity in cortico-striato-limbic regions (Yang et al., 2010, 2015; Ping et al., 2013; Chen et al., 2016; Niu et al., 2017). One important limitation of these studies is the small sample sizes, which reduce sensitivity and presumably result in the lack of reliability of findings. Additionally, all these published mass-univariate researches investigating ReHo alterations between OCD participants and healthy control subjects (HCS) aim to test whether there are any effects in one or more brain regions, rather than to test whether the effects are large enough to have the translational importance for clinical utility.

Recently, the researchers have developed a growing interest in applying multivariate pattern analysis (MVPA) to develop brain signatures for clinical diagnoses in relevant mental disorders (Woo et al., 2017). Relative to traditional univariate analysis, the MVPA bears two strengths. First, MVPA takes the inter-correlation between voxels into consideration and thus might be more sensitive in detecting subtle and spatially distributed alterations. Second, MVPA allows statistical inferences at the single-subject level and thus could be used to make diagnostic decisions of individual patients (Vieira et al., 2017). Meanwhile, MVPA methods have been successfully used to differentiate OCD participants from control subjects based on diffusion MRI (Li et al., 2014), structural MRI (Hu et al., 2016), as well as task-based fMRI (Weygandt et al., 2012). However, no MVPA studies have investigated the utility of ReHo maps for distinguishing OCD subjects from HCS.

Therefore, we aimed to apply both univariate analysis and support vector machine (SVM) method to evaluate the alterations of ReHo in a relatively large sample of medication-free patients with OCD. The SVM discrimination algorithm is a widely used MVPA approach. In the SVM analysis, the data points are first projected into the high-dimensional space and subsequently classified based on the principle of maximizing the margin between categories (Orrù et al., 2012). The SVM classification process constitutes two procedures: training and testing. In the training stage, a decision boundary is identified by the SVM classifier to separate the examples into the input space according to their class labels (i.e., OCD vs. HCS). In the testing phase, as soon as the optimized hyperplane is established from the training dataset, it can be utilized to make predictions for the class label of a new testing example to determine its generalizability (Noble, 2006). We hypothesized that the SVM analysis of the ReHo maps: (1) would potentially be able to discriminate individual patients with OCD from healthy controls; and (2) provided information on neurobiological changes that will potentially help to elucidate the mechanisms that cause OCD.

## Materials and Methods

### Participants

All the subjects were right-handed individuals with ages between 18 and 60 years. Patients with OCD were enrolled from the West China Hospital of Sichuan University. The diagnosis of OCD was determined by three experienced clinical psychiatrists (YY, BL, and WT with 32, 18, and 11 years of experience in clinical psychiatry, respectively) by using the Structured Clinical Interview for *Diagnostic and Statistical Manual of Mental Disorders, Fourth Edition* (*DSM-IV*; Patient Edition) to exclude anxiety disorder, Tourette syndrome, major depressive disorder, schizophrenia, bipolar disorder, or any other axis I psychiatric comorbid disorders. The symptom severity of OCD was rated according to the Yale–Brown Obsessive Compulsive Scale (Y-BOCS). The anxiety symptom severity was assessed by the 14-item Hamilton Anxiety Scale (HAMA). The depressive symptom severity was evaluated based on the 17-item Hamilton Depression Scale (HAMD). All the patients with OCD experienced a washout period of 4 weeks from any treatment before the image data acquisition. Healthy controls were recruited by a poster and were screened using the structured clinical interview for DSM-IV axis I disorders (SCID) (non-patient edition) in order to ensure the absence of neurological and mental diseases. All the HCS reported that their first-degree relatives did not have a history of mental illness or neurological diseases. The exclusion criteria applied to both OCD patients and HCS were listed as follows: (1) the existence of neurological diseases or other mental disorders; (2) any history of cardiovascular diseases, metabolic disorders, or major physical illness; (3) alcohol or drug dependance; and (4) pregnancy. The above assessments were evaluated by two experienced psychiatrists. Written informed agreement was obtained from each participant before the research procedure was initiated. The Ethics Committee of West China Hospital approved the present study.

### Resting-State Functional Magnetic Resonance Imaging Data Acquisition

All participants were subjected to MRI scanning using a 3.0-T GE Signa EXCITE scanner with an 8-channel phase array head coil. The RS-fMRI sensitized to alterations in BOLD signal levels of the whole brain was obtained *via* an echo-planar imaging (EPI) sequence. The parameters of scanning were listed as follows: repetition time (TR) = 2,000 ms, echo time (TE) = 30 ms, flip angle = 90°, slice thickness = 5 mm with no slice gap, field of view (FOV) = 240 × 240 mm^2^, 30 axial slices, and 200 time points in each run. In the MRI examination, participants were informed to be relaxed and keep their eyes closed without falling asleep. Foam padding was used to reduce head motion while earplugs were used for reducing the scanner noise.

### Image Preprocessing

The image preprocessing procedures including slice timing, head motion correction, and normalization (voxel size = 3 × 3 × 3 mm^3^) to Montreal Neurological Institute (MNI) space were performed using the data processing and analysis for (resting-state) brain imaging (DPABI) software (Yan et al., 2016). For the purposes of removing head motion artifacts, we applied the Friston 24-parameter model in the current study as a regressor, which has been shown to be advantageous to the six-parameter model (Yan et al., 2013). All the image data used in the present study met the criteria of spatial movement in any direction <1.5 mm or 1.5° and the mean framewise displacement (FD) value <0.2. Signal from the cerebrospinal fluid, white matter, and global mean signal intensity was used as covariates for decreasing the effects of non-neuronal BOLD fluctuations. Afterward, we removed the linear trend of the fMRI images, and band-pass filtering (0.01–0.08 Hz) was performed in order to reduce the effect of physiological noise with high frequency as well as the extreme low-frequency drift. After that, we adopted the REST software (Song et al., 2011) for the calculation of the ReHo maps.

### Regional Homogeneity Calculation

The ReHo map of each subject was created by the calculation of KCC regarding the time series between a single voxel with adjacent 26 voxels of its neighbors in a voxel-wise manner (Zang et al., 2004). Subsequently, the REST software used a whole-brain mask to eliminate the non-brain tissues for the aims of standardization, Afterwards, individual ReHo maps were divided using the average global KCC, and this procedure was achieved in the whole-brain mask. Finally, a Gaussian kernel with a full width at half maximum of 8 mm was adopted to spatial smoothing for all the individual standardized ReHo maps.

### Univariate Analysis

We performed a univariate analysis for exploring alterations of localized connectivity by comparing ReHo maps between patients with OCD and HCS using the two-sample *t*-test in SPM8. Meanwhile, we conducted subgroup analysis in medication-naive OCD patients compared with matched HCS to test the reliability of the main effects. The voxel-level statistical threshold was set at *P* < 0.001 with a minimum cluster extent of 100 voxels without correction. Meanwhile, the statistical threshold of cluster level was set at *P* < 0.05 with family-wise error (FWE) correction. Regions with significant ReHo alterations between groups were extracted as region of interest for Pearson correlation analyses with clinical variables including illness duration, HAMA scores, HAMD scores, and symptom severity evaluated by Y-BOCS and subscale scores in the SPSS software (SPSS 16.0; Chicago, IL, USA).

### Multivariate Pattern Analysis Approach

The SVM classifier was adopted to evaluate the classification accuracy of local connectivity in distinguishing individuals with OCD from HCS, and this step was conducted by the PROBID package running in the Matlab. The SVM approaches have been described in detail elsewhere (Li et al., 2014; Hu et al., 2016) and are briefly summarized here. Individual RS-fMRI images were regarded as points situated in a high-dimensional interspace defined by the ReHo maps in the preprocessed images. The SVM classifier identified a linear decision boundary to separate individual brain maps in a high-dimensional space based on the category label. The optimized hyperplane was computed on the basis of the whole multivariate pattern of the ReHo map across each RS-fMRI scan. For the purposes of lowering the risk of data overfitting and allowing direct extraction of the weight vector, the linear kernel SVM was applied. Additionally, we utilized a leave-one-out cross-validation (LOOCV) strategy to evaluate the performance of the SVM classifier. This LOOCV approach excluded an independent individual from each group and used the rest of the participants to train the SVM classifier. Then, the excluded subject pair was applied to test the differentiating ability of SVM to reliably discriminate between two sorts. We used a nonparametric test to determine the statistical significance of the general accuracy of discrimination, specifically, the nonparametric test repeated the classification step 1,000 times using permutation test of the labels in the training group and computed the specificity and sensitivity in respect to the true labels. Ultimately, the PROBID software generated the discriminative maps to display the relative contributions of each voxel to the SVM classification decision. We overlaid the voxels with top 30% of the maximum weighted value onto the brain template with high resolution (Mourão-Miranda et al., 2005).

## Results

A total of 88 participants with OCD and 88 gender- and age-matched control subjects were employed in the current study. Among them, 54 OCD patients and 54 control subjects were included in a previous analysis (Bu et al., 2019). Table 1 displays the clinical characteristics and demographic information of all the subjects. No significant differences were identified with respect to gender (*P* = 1.000) and age (*P* = 0.381) between OCD patients and HCS. The mean (standard deviation) scores of Y-BOCS, HAMD, and HAMA were 21.47 (±5.38), 8.74 (±4.92), and 8.78 (±4.46), respectively. The illness duration of the OCD patient group was 7.32 ± 5.58 years.

**Table 1:** Clinical characteristics and demographic information for patients with obsessive-compulsive disorder (OCD) and healthy control subjects (HCS).

Characteristic	OCD (*n* = 88)		HCS (*n* = 88)		Significance	
	Mean	SD	Mean	SD	*t*/*χ*^2^	*P*
Gender (male:female)	56:32	-	56:32	-	0.000	1.000
Age (years)	29.16	8.71	27.88	10.58	0.879	0.381
Duration of illness (years)	7.32	5.58	-	-	-	-
Age of onset	21.84	7.09	-	-	-	-
Y-BOCS total	21.47	5.38	-	-	-	-
Obsessions	13.15	5.07	-	-	-	-
Compulsions	8.32	5.35	-	-	-	-
HAMD 17	8.74	4.92	-	-	-	-
HAMA 14	8.78	4.46	-	-	-	-
Current treatment status	*n*	%				
Drug-free (>4 weeks)	88	100	-	-	-	-
Medication-naive	74	88.09	-	-	-	-
Previous treatment history	*n*	%	-	-	-	-
Clomipramine	4	4.55	-	-	-	-
Paroxetine	3	3.41	-	-	-	-
Fluoxetine	3	3.41	-	-	-	-
Sertraline	3	3.41	-	-	-	-
Quetiapine	1	1.14	-	-	-	-

*Abbreviations: HAMA, Hamilton Anxiety Rating Scale; HAMD, Hamilton Depression Rating Scale; HCS, healthy control subjects; OCD, obsessive-compulsive disorder; SD, standard deviation; Y-BOCS, Yale–Brown Obsessive Compulsive Scale*.

**Figure 1:**
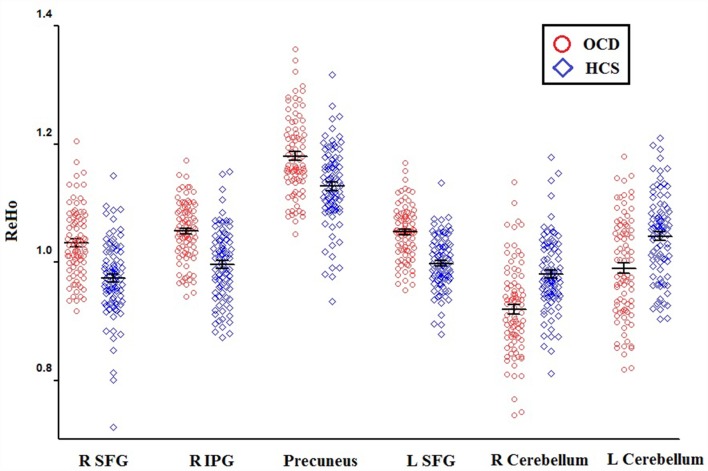
The distributions of ReHo eigenvalue in six brain areas with altered ReHo in OCD patients relative to HCS. Abbreviations: HCS, healthy control subjects; IPG, inferior parietal gyrus; OCD, obsessive-compulsive disorder; ReHo, regional homogeneity; SFG, superior frontal gyrus.

**Figure 2:**
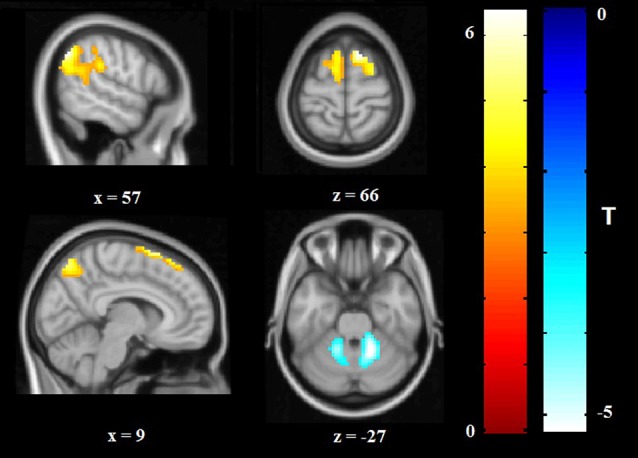
Altered ReHo in OCD patients compared with HCS. ReHo increases are indicated in warm colors while ReHo reductions are indicated in cool colors. Abbreviations: HCS, healthy control subjects; OCD, obsessive-compulsive disorder; ReHo, regional homogeneity.

**Figure 3:**
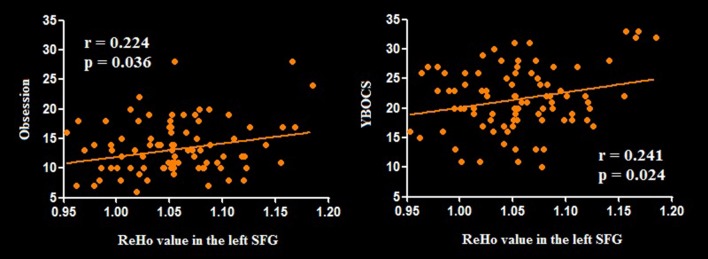
Pearson correlation exhibiting positive association between ReHo in the left SFG and YBOCS as well as obsessive subscale scores in the OCD group. Abbreviations: OCD, obsessive-compulsive disorder; ReHo, regional homogeneity; SFG, superior frontal gyrus; YBOCS, Yale–Brown Obsessive Compulsive Scale.

Relative to HCS, OCD patients showed lower ReHo in the bilateral cerebellum and higher ReHo in the bilateral superior frontal gyri (SFG), right IPG, and precuneus (*P* < 0.05, with FWE correction at the cluster level; Table 2, Figures 1, 2). The results regarding subgroup analysis of medication-naive OCD patients remained reproducible with the main effect (Supplementary Figure S1). The ReHo value in the left SFG positively correlated with Y-BOCS total score (*r* = 0.241, *P* = 0.024) and obsessive subscale (*r* = 0.224, *P* = 0.036; Figure 3). No significant correlations were identified between altered ReHo and any other clinical characteristics in OCD patients.

**Table 2:** Significant differences of regional homogeneity (ReHo) alterations in OCD patients compared with HCS.

Region	Side	Voxel size	MNI coordinate			*T*	P*
			*x*	*y*	*z*		
**OCD patients > HCS**							
Superior frontal gyrus	R	274	15	15	66	6.35	0.001
			15	27	60	5.55	
			9	9	69	5.37	
Inferior parietal gyrus	R	407	57	−60	39	6.25	<0.001
			63	−54	33	5.46	
			63	−54	24	5.39	
Precuneus	L/R	139	9	−69	57	5.13	0.018
			−9	−78	48	3.33	
			−6	−78	39	3.20	
Superior frontal gyrus	L	591	−12	0	72	5.10	<0.001
			−42	6	54	5.07	
			−48	36	12	5.06	
**OCD patients < HCS**							
Cerebellum	R	357	15	−51	−27	−5.20	<0.001
			6	−27	−15	−3.86	
Cerebellum	L	117	−18	−48	−27	−4.55	0.031
			−9	−60	−21	−4.04	

*P < 0.05 set at cluster level with whole-brain family-wise error correction. *Abbreviations*: HCS, healthy control subjects; MNI, Montreal Neurological Institute; OCD, obsessive-compulsive disorder; ReHo, regional homogeneity.

**Figure 4:**
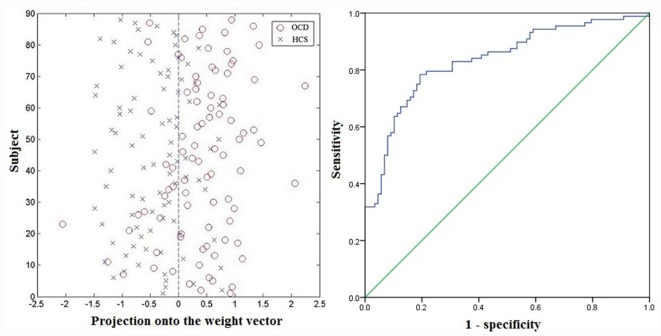
Classification plot (left) and receiver operating characteristic (ROC) curve (right) for differentiating patients with OCD from HCS based on ReHo maps, yielding an accuracy of 78.98% (sensitivity = 78.41%, specificity = 79.55%, *P* < 0.001). Abbreviations: HCS, healthy control subjects; OCD, obsessive-compulsive disorder; ReHo, regional homogeneity.

The plotting for SVM classification using ReHo maps are presented in Figure 4 (left). The receiver operating characteristic (ROC) curve assessing the performance of the SVM classifier based on ReHo maps is displayed in Figure 4 (right).

**Figure 5:**
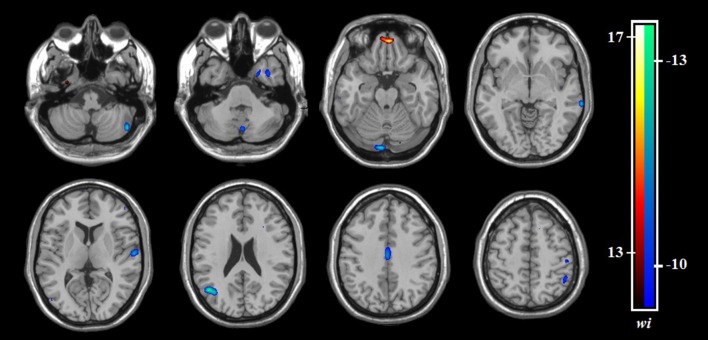
The discrimination maps for ReHo. These areas were identified by setting the threshold to the top 30% of weight vector scores. Warm colors indicated higher discriminated values for OCD subjects than HCS. Cool colors indicate lower discriminated values for OCD participants than HCS. Abbreviations: HCS, healthy control subjects; OCD, obsessive-compulsive disorder; ReHo, regional homogeneity.

Based on the SVM classification approach, the diagnostic accuracy of ReHo maps for the contrast between OCD and HCS was 78.98% (sensitivity = 78.41% and specificity = 79.55%, *P* < 0.001). The most discriminative regions contributing to the SVM classification regarding ReHo were mainly located in frontal, temporal, and parietal regions as well as the cerebellum while the right OFC was identified with the highest discriminative power. The details concerning the brain areas that contributed to distinguishing OCD participants from controls are displayed in Table 3 and Figure 5.

**Table 3:** Brain areas that differentiated OCD patients from HCS based on ReHo maps.

Brain areas (SVM)	Coordinates (MNI)			*w*_i_
	*x*	*y*	*z*	
**OCD patients > HCS**				
R orbit frontal	5	53	−19	17.18
**OCD patients > HCS**				
R cerebellum	50	−67	−46	−12.88
Vermis	5	−73	−37	−11.39
R inferior temporal	38	2	−40	−11.01
R middle temporal	23	2	−40	−10.88
R middle temporal	71	−34	−4	−12.69
R transverse temporal	62	−13	8	−12.28
L middle temporal	−45	−64	23	−13.26
Dorsal anterior cingulate	1	−13	38	−11.42
R inferior parietal	50	−46	54	−10.57

*These areas were identified by setting the threshold to the top 30% of the weight vector scores. Abbreviations: HCS, healthy control subjects; MNI, Montreal Neurological Institute; OCD, obsessive-compulsive disorder; ReHo, regional homogeneity; SVM, support vector machine; w_i_, weight of each cluster centroid i*.

## Discussion

The current study applied both univariate analysis and MVPA approach to explore the alterations of ReHo in a relatively large cohort of medication-free patients with OCD. Our study revealed that OCD patients showed lower ReHo in the bilateral cerebellum and higher ReHo in the frontoparietal regions compared with HCS. ReHo value in the left SFG positively correlated with symptom severity. Additionally, OCD patients could be differentiated from HCS using SVM based on ReHo maps with high classification accuracy (78.98%, *P* < 0.001). Brain regions including the frontolimbic circuit, the temporo-parietal areas, and the cerebellum were identified to have high differentiating power. Meanwhile, both univariate analysis and MVPA approach detected abnormalities of frontoparietal regions and cerebellum in OCD based on ReHo maps.

The PFC has long been recognized to be a paramount part in the mediation of clinical manifestations including executive disturbance, low behavioral flexibility, and inability for decision making that are commonly observed in OCD patients (Chamberlain et al., 2008; Menzies et al., 2008; Samara et al., 2017). Furthermore, Ahmari et al. (2013) used optogenetics to prove that repeated stimulation of the OFC in mice would lead to persistent OCD-like behaviors. In agreement with findings from previous ReHo studies of OCD (Ping et al., 2013; Yang et al., 2015; Chen et al., 2016), the current study revealed higher ReHo in the bilateral SFG in OCD patients compared with HCS. Meanwhile, the right OFC was identified as the brain region that provided the greatest potential to discriminate OCD from HCS based on the MVPA approach. Thus, an abnormal hyperactivation in PFC might be related to the pathophysiological process of OCD. Additionally, the increased ReHo value in the left SFG was positively correlated with the Y-BOCS and obsession subscale scores. This finding indicated that ReHo might be an optimal measure for capturing the disease severity.

Neither univariate analysis nor MVPA revealed ReHo alterations of the striatum in OCD patients, which is inconsistent with evidence from meta- and mega-analyses of structural and functional neuroimaging studies (Menzies et al., 2008; Radua and Mataix-Cols, 2009; Rotge et al., 2010; Boedhoe et al., 2017). The striatum has long been regarded as a key hub for symptom mediation in OCD (Pauls et al., 2014) and recent evidence has suggested the striatum as a promising site for deep brain stimulation treatment of OCD (Pinhal et al., 2018). Two previous studies identified that OCD patients showed decreased ReHo in the caudate nucleus (Ping et al., 2013; Yang et al., 2015), which seemed to be inconsistent with the present finding. Several reasons might account for the discrepancies. First, there were more OCD participants enrolled in the present study than in the previous ReHo studies (Ping et al., 2013; Yang et al., 2015). The large sample size of the current study increased the power to detect potential localized functional disruptions in OCD and minimized the risk of false-positive findings. Second, clinical cohorts in previous ReHo studies included OCD patients who were being treated using serotonin reuptake inhibitors while all the subjects with OCD of our research were medication-free. This is especially relevant, given the report by Beucke et al. (2013) that antidepressant medication may reduce network-level neural function of cortico-striato-thalamo-cortical (CSTC) circuits in OCD. Third, two recent multicenter studies of 780 brain scans reported no structural alterations of striatum in OCD patients (de Wit et al., 2014; Fouche et al., 2017). Therefore, striatum may not directly relate to the pathophysiology of OCD, but be secondary to current medication use. Further longitudinal studies would be needed to clarify this hypothesis.

Both univariate analysis and MVPA demonstrated cerebellar dysfunction of OCD patients compared with healthy controls. The cerebellum has been identified to integrate the information flow of prefrontal-basal ganglia pathway as it is functionally and anatomically connected to the CSTC network (Middleton and Strick, 2000). Furthermore, an increasing number of evidence has reported that, besides the traditional role of motor control, the primate cerebellum is involved in cognitive control and emotional regulation (Ramnani, 2006). For example, the cerebellar hypoactivation in OCD patients was observed when fear conditioning tasks were performed (Milad et al., 2013), which was consistent with our current finding. Interestingly, a recent multicenter voxel based morphometry (VBM) mega-analysis reported smaller volumes of PFC and greater cerebellar gray matter volume bilaterally (de Wit et al., 2014), but our study found higher ReHo in the bilateral SFG and lower ReHo in the bilateral cerebellum. The opposite findings might suggest a functional compensatory response to regional anatomical alterations in OCD. Given all the OCD participants were treatment-free in the current study, we proposed that the activation disequilibrium between the PFC and the cerebellum might directly be associated with the psychopathology of OCD.

In addition to the significant cerebellar dysfunction, it is of special interest to find the involvement of parietal (including IPG and precuneus) and temporal regions with disrupted localized connectivity in patients with OCD compared to healthy volunteers, which is consistent with the reports of the engagement of the extended OCD anatomical model (Gürsel et al., 2018). Previous systematic review of structural neuroimaging studies had emphasized that the neural mechanism of OCD might involve more widespread regions such as parietal and temporal cortices, which may help explain the heterogeneity in clinical manifestations of OCD (Piras et al., 2013). By integrating evidence from functional neuroimaging and neuropsychological tests, Menzies et al. (2008) suggested the parietal cortex as a fundamental area outside the classical orbitofronto-striatal circuit in the psychopathology of OCD. Moreover, altered anatomical connectivity between lateral frontal and parietal regions is consistently identified in the meta-analysis of diffusion tensor imaging studies (Piras et al., 2015). The parietal activation may be related to emotion processing (Margulies et al., 2009) and decision making (Huk et al., 2017), which are also reported to be impaired in OCD patients. Meanwhile, de Vries et al. (2014) suggested that the compensatory activity of frontoparietal network (FPN) during working memory might constitute a neurocognitive endophenotype for OCD. The precuneus and temporal cortices are major components of the default mode network (DMN; Raichle, 2015). A recent task-based fMRI meta-analysis has demonstrated that OCD symptom severity was related to increased activation in the precuneus (Thorsen et al., 2018). Evidence from cognitive studies has demonstrated that temporal regions are involved in impairment of inhibitory control (Penadés et al., 2007) and executive planning (van den Heuvel et al., 2005) in OCD. Research using graph theory analysis revealed decreased local efficiency and reduced intra-connectivity of DMN in OCD patients relative to HCS (Peng et al., 2014). Since the IPG is reported as an important node in both the FPN and the DMN (Boedhoe et al., 2018) while our present study identified higher ReHo in the IPG, we speculated that the parietal hyperactivation might contribute to the underlying mechanism of OCD.

The current research had some limitations. First, although our results were encouraging, multicenter research is still needed for testing the generalizability of the current findings. Second, we recruited drug-free OCD patients who went through a 4-week medication washout period before the MRI data acquisition. Therefore, we could not rule out the longer-term effects of treatment on the localized functional disruptions. Third, we only compared patients with OCD and HCS in the current investigation, Thus, it remained unknown whether the SVM classification of ReHo maps would distinguish OCD subjects from participants with different psychiatric disorders. Future investigations could address this issue by evaluating the discrimination accuracy of SVM classifier in differentiating OCD from other anxiety disorders (i.e., Tourette syndrome). Fourth, we did not recruit pediatric OCD patients in this study. Recent studies have identified distinct cortical and subcortical alterations in pediatric and adult OCD (Boedhoe et al., 2017, 2018; Hu et al., 2017). Future studies should involve pediatric OCD patients in order to provide an insight into the neurodevelopmental alterations in this disorder. Fifth, we did not evaluate the possible effect of comorbid apathy in the current study. Previous evidence indicated that apathy could modulate the microstructure of bundles related to OCD (Spalletta et al., 2013). Future investigations should adopt the Apathy Rating Scale to assess the apathetic conditions in OCD patients. Finally, we failed to perform separate analyses for childhood onset OCD and adulthood onset OCD since there were much fewer childhood onset OCD patients (*n* = 18) than adulthood onset OCD patients (*n* = 70). A recent world-wide mega-analysis reinforced the effects of onset age in the neural mechanism of OCD (Boedhoe et al., 2017). Thus, illustrating the differences of ReHo alterations between childhood onset OCD patients and adulthood onset OCD patients is still warranted.

In summary, our univariate analysis provided evidence that localized activation disequilibrium between the PFC and the cerebellum appeared to be associated with the pathophysiology of OCD. Additionally, positive correlations between ReHo in left SFG and symptom severity were identified, which suggested that ReHo might be a paramount measure for studying the underlying cause of OCD. Furthermore, the SVM analysis of ReHo achieved modest accuracy (79.0%, *P* < 0.001) in classifying OCD patients and HCS at the individual level, which indicated the translational role of the localized connectivity as a potential psychoradiological biomarker for OCD diagnosis.

## Ethics Statement

The study was approved by the Research Ethics Committee of the West China Hospital, Sichuan University, and written informed consent was obtained from each participant.

## Author Contributions

XQH, QG, and YY designed the study. XYH, BL, and WT acquired the data. XYH, LZ, XB, and HL analyzed the data. XQH, NR, XYH, and QG wrote the article, which BL, WT, LZ, XB, HL, LL, XXH, ST, YG, and YY reviewed. All authors approved the final version for publication.

## Conflict of Interest Statement

The authors declare that the research was conducted in the absence of any commercial or financial relationships that could be construed as a potential conflict of interest.
