# Supplementary material for: Localized Connectivity in Obsessive-Compulsive Disorder: An Investigation Combining Univariate and Multivariate Pattern Analyses
Source: Front Behav Neurosci. 2019 Jun 13;13:122. doi: 10.3389/fnbeh.2019.00122 (PMC6584748; doi:10.3389/fnbeh.2019.00122)

***Supplementary Online Content***

**Localized connectivity in obsessive-compulsive disorder: An investigation combining univariate and multivariate pattern analyses**

Xinyu Hu, Lianqing Zhang, Xuan Bu, Hailong Li, Bin Li, Wanjie Tang, Lu Lu, Xiaoxiao Hu, Shi Tang, Yingxue Gao, Yanchun Yang, Neil Roberts, Qiyong Gong and Xiaoqi Huang

**Figure S1.** Subgroup analysis of altered ReHo in medication-naive OCD patients (N = 74) compared with HCS (N = 74). ReHo increases are indicated in warm colors while ReHo reductions are indicated in cool colors.

*Abbreviations:* HCS, healthy control subjects; OCD, obsessive-compulsive disorder; ReHo, regional homogeneity.

**Figure S1.** Subgroup analysis of altered ReHo in medication-naive OCD patients (N = 74) compared with HCS (N = 74). ReHo increases are indicated in warm colors while ReHo reductions are indicated in cool colors.

*Abbreviations:* HCS, healthy control subjects; OCD, obsessive-compulsive disorder; ReHo, regional homogeneity.


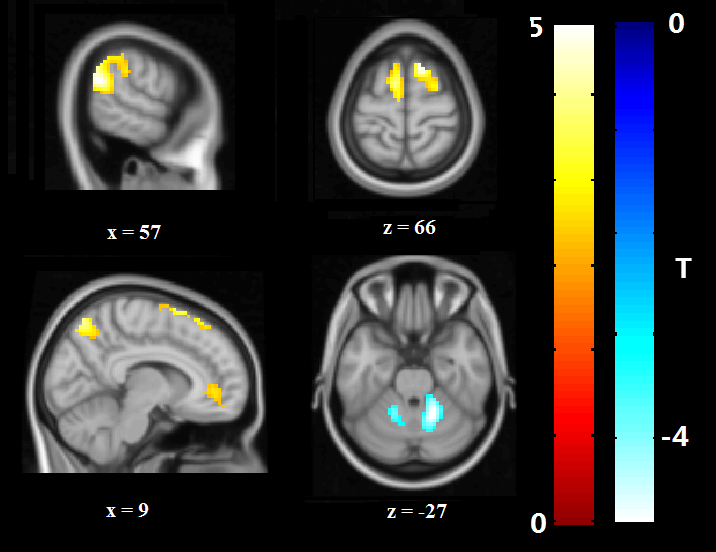

Supplement: Supplementary file 1 [file Table_1.DOCX]
